# 

**Published:** 1873-01

**Authors:** 


THREE DOLLARS A YEAR. SPECIMEN COPIES, 30 CENTS.

VOL. XXX. January, 1873. NUMBER 1.

THE

### Chicago Medical Journal.

J. ADAMS ALLEN, M.D., LL.D., WALTER HAY, M.D.,

EDITORS.

CHICAGO:

W. B. KEEN, COOKE & CO., PUBLISHERS,

NOS. 113 AND 115 STATE STREET.

*The postage on this Journal is 24 cents a year—payable quarterly in advance.*

